# Physical fitness and physical self-concept of male and female young adults in Qatar

**DOI:** 10.1371/journal.pone.0223359

**Published:** 2019-10-10

**Authors:** Bryna C. R. Chrismas, Lina Majed, Zsuzsanna Kneffel

**Affiliations:** Qatar University, Sport Science Program, College of Arts and Science, Doha, Qatar; Teesside University/Qatar Metabolic Institute, UNITED KINGDOM

## Abstract

**Background:**

Physical inactivity is high within the Qatari population, particularly within females, and school-based environments, contributing to increased morbidity and mortality. School-based physical activity (PA) outcomes may be mediated by physical self-concept. Low physical self-concept may negatively impact PA engagement, compromising childhood and adolescent physical fitness, which may translate into adulthood. Normative physical fitness data for the Qatari population is unavailable. Stratifying normative physical fitness appears prudent, to not only allow comparisons to be made worldwide, but enable informed decisions for public health policy and future interventions in the Qatari population.

**Purpose:**

To establish the physical fitness of young adults in Qatar, and examine differences between males and females for physical self-concept, and engagement in school-based and extra-curricular PA.

**Method:**

186 (females n = 85) healthy participants [median (minimum—maximum) age: males = 21 (18–26), females = 21 (18–24) y; height: males = 1.74 (1.57–1.99), females = 1.61 (1.46–1.76) m; body mass: males = 71.9 (49.3–145.0), females = 56.8 (35.7–96.4) kg] completed the ALPHA-FIT test battery for adults (one leg stand, figure of eight run, handgrip strength, jump and reach, modified push-up, dynamic sit-up and 2 km walk), physical self-description questionnaire (measuring physical self-concept), and were asked to answer ‘yes’ or ‘no’ to whether they participated in school-based and extra-curricular PA.

**Results:**

Data is reported as effect size; ±90% confidence limit. Males compared to females *most likely* performed better for dynamic sit-up (2.2; ±0.76), *very likely* better for the figure of eight run (0.86; ±0.42) and *likely* better for handgrip strength (2.1; ±0.75). Males *likely* had higher physical self-concept for coordination (0.78; ±0.37) and endurance (0.66; ±0.27) compared to females. There were no differences for school-based PA (p ≥ 0.78) or for extra-curricular PA for males (p ≥ 0.26) or females (p ≥ 0.21).

**Conclusion:**

The data suggests that the young Qatari adult population has variable, yet generally low, physical fitness traits compared to individuals worldwide, likely due to their low PA. The precise aetiology for this is not well documented, yet such data may be prudent to evidence-inform strategies to improve physical fitness through increased PA (synergistic relationship), given the strong association between physical activity/fitness and morbidity/mortality.

## Introduction

Within Qatar, 83% of the population participate in little or no physical activity (PA) whilst 63% engage in no PA whatsoever [1]. This generates estimated annual direct (health-care expenditure) and indirect (productivity losses) costs of $60.7 million [2]. Physical inactivity increases risk of heart disease, stroke, diabetes and certain cancers (e.g. breast and colon), and is one of the leading risk factors for death worldwide [3]. It is concerning therefore that only 39% of Qatari school children (6–12 y) meet the school-based moderate-to-vigorous PA guidelines of ≥ 30 min per day [4]. Furthermore, the Qatar Active Healthy Kids Report Card, which assesses physical activity in children and youth (6–17 y) utilising a grading system [A+ to F, or ‘incomplete’ (inadequate information to assign a grade)] revealed poor grades specifically for sedentary behaviour (D+), overall PA (D), and physical fitness (incomplete) [5]. This resulted in Qatar placing 20^th^ out of 30 for high Human Development Index countries and regions [6]. Qatar has made demonstrable improvement since the first Report Card in 2016 [7]. Nevertheless, the overall results are still extremely concerning, particularly as physical fitness was graded as incomplete in 2016 and 2018. Low physical fitness during childhood is an extremely powerful marker of health, and is associated with increased risk of obesity and cardiovascular disease, poor mental health and increased mortality [8]. Concerningly, it appears physical fitness of children and adolescents is declining worldwide [9–13], which may further compound disease risk and quality of life in adulthood [14–17]. Worldwide, girls typically perform worse in cardiorespiratory fitness and strength [12, 18–20], which longitudinally are predictors for metabolic syndrome [18]. Given that childhood and adolescence are key periods of life, and often the times at which lifestyle behaviours (i.e. physical activity) are established [21], it is essential that school-based PA provides the appropriate quantity and quality of PA to improve physical fitness. School-based PA outcomes may be mediated by physical self-concept. Such psychological constructs may impact the engagement and outcomes associated with school-based PA [22], compromising physical fitness of children and adolescents, which may translate into adulthood [23].

Physically active adults possess higher physical fitness [9, 10] and demonstrate lower morbidity and mortality [11]. Muscular and cardiorespiratory fitness are essential components of physical fitness. Indeed, high cardiorespiratory and muscular fitness have been shown to be cardioprotective, irrespective of body mass index (BMI) [24–26]. Additionally, muscular fitness is inversely associated with metabolic risk, and mortality, independent of cardiorespiratory fitness [27, 28]. Specifically, push-up capacity [29] and handgrip strength [30, 31] are inversely associated with adverse cardiovascular disease events, and all-cause mortality [30, 32]. However, no objective physical fitness data currently exists for the Qatari population, unlike Europe for example, where age stratified fitness data are being established [18, 33–35]. Furthermore, worldwide (e.g. Australia, UK, USA, Canada, Japan, Norway, Sweden) age and gender stratified normative data for handgrip strength [36–43], and push-up capacity [44–47] exist. Obtaining simple, cost-effective and objective measures of physical fitness (i.e. push-ups, handgrip strength) appears essential for the Qatari population, as this would allow direct comparison to existing worldwide normative data. Moreover, establishing a longitudinal normative database of these measures for the Qatari population is an important first step for evidence-informed decisions to be made, in order to improve physical fitness, and therefore, attenuate morbidity and mortality within the Qatari population. Such data will likely demonstrate gender differences, as 44% of Qatari females achieve < 5,000 steps per day [48], likely a combination of the environment (i.e. heat, humidity, dust), culture, and Islamic traditional clothing (i.e. Abaya, Hijab), adopted widely by Qatari females in public places [49]. Additionally, Arab females report poorer sport, physical, and strength components of physical self-concept compared to their male counterparts [50]; important as physical self-concept is positively associated with PA engagement [51] and motor skill development [52]. However, no data currently exists examining gender differences in both objective physical fitness and physical self-concept within the Qatari population.

Therefore, the primary aim of this study was to characterize physical fitness and physical self-concept in young adults in Qatar. A secondary aim was to examine differences in physical fitness and physical self-concept for gender, and engagement in school-based or extra-curricular PA. It was hypothesised that (i) males would demonstrate superior physical fitness and physical self-concept compared to females, and (ii) that physical fitness would be higher in individuals that engaged in both school-based and extra-curricular PA.

## Methods

### Experimental design

Ethical approval for this cross-sectional study was received from Qatar University Institutional Review Board (QU-IRB 428-E/15). Prior to any experimental procedure occurring, written informed consent was obtained in the spirit of the Declaration of Helsinki (1975). All testing was performed at Qatar University within the male and female indoor sports hall. Participants performed the tests individually, and all tests were performed on the same day. Testing occurred between October 2015 and February 2016. The physical activity and health questionnaire provided in the ALPHA-FIT manual for adults [53] was completed prior to any testing. The following pre-test procedures were followed; avoidance of severe physical exertion 48-h prior to testing, avoidance of physical exertion on the day of testing, a minimum of 8-h sleep prior to testing, last meal 3-h prior to testing, no smoking or caffeinated beverages 1-h prior to testing [53]. Additionally, participants were asked to answer ‘yes’ or ‘no’ to whether they participated in school-based and extra-curricular PA. Examples of what constituted school-based PA (i.e. physical education classes, sports day), and extra-curricular (i.e. schools Olympic program, swimming lessons, team/individual sports clubs) were provided to the participants.

### Participants

A total of 186 (females n = 85) healthy participants volunteered for this study. Participant characteristics are shown in Table 1. A convenience sample was recruited via word-of-mouth, email and social media by students from Qatar University Sport Science Program. Eligible participants were born in Qatar, completed elementary and high-school in Qatar, were aged between 18–29 y and were not taking any medication. Exclusion criteria included cardiorespiratory disease and/or symptoms, chest pain or breathing problems at rest or during exercise, hypertension (≥ 140/90 mmHg), light headiness and/or dizziness, inflammatory joint disease, back problems or other long-term and/or repetitive musculoskeletal problems [53].

**Table 1:** Participant characteristics. Data is reported as median (minimum–maximum).

	Males	Females
Age (y)	21 (18–26)	21 (18–24)
Height (m)	1.74 (1.57–1.99)	1.61 (1.46–1.76)
Body mass (kg)	71.9 (49.3–145.0)	56.8 (35.7–96.4)
Systolic blood pressure (mmHg)	118 (96–139)	117 (91–136)
Diastolic blood pressure (mmHg)	80 (52–85)	74 (56–85)

### Physical self-concept

All participants completed the short version of the physical self-description questionnaire (PSDQ-S), scored from 0 to 6 on a Likert scale. The PSDQ-S is comprised of nine specific self-concept scales (activity, appearance, body fat, coordination, endurance, flexibility, health, sport, and strength), and two global scales (global physical and global esteem), and has shown good validity across a heterogeneous sample, including Arab university students [54]. Each PSDQ-S item (40 items in total) is a simple declarative statement (e.g. ‘I am good at coordinated movements’). Participants responded to each PSDQ-S item using the 6-point true-false response scale (1 = false, 2 = mostly false, 3 = more false than true, 4 = more true than false, 5 = mostly true, 6 = true). Each of the 40 items is denoted by three codes comprising of numbers and letters which were used for scoring. Simple scores for each of the nine specific scales (detailed above) were calculated by summing responses to items from each scale and dividing by the number of items. Negatively worded items (those denoted by * in the scoring instructions) were reversed scored before summing the responses. The PSDQ-S provides a concentrated view of physical self-concept, and is therefore considered more appropriate for sport and exercise research [54]. The PSDQ-S is considered to be psychologically robust, and has demonstrated good reliability and high construct validity [22].

### Anthropometric and baseline measures

Anthropometric measures were obtained using the KaWe wall mounted stadiometer (Kirchner & Wilhelm GmbH & Co. KG, Germany), and OMRON (OMRON Healthcare Europe B.V., Netherlands) scales for body mass following the international society for the advancement of kinanthropometry standard procedures. Blood pressure was measured using a fully automated device (OMRON M3 comfort, OMRON Healthcare Europe B.V., Netherlands), following standard procedures (5-min rest in a seated position, with the back supported and the arm resting on a table at the level of the heart).

### ALPHA-FIT test battery

The ALPHA-FIT test battery for adults aged 18–69 y was employed to measure seven field-based physical fitness components. As per the instruction manual [53], no warm-up or stretching was performed prior to testing. Practice trials were allowed as written in the test battery (please see Table 2), and when a second test trial was performed there was a 30 s rest period between trials (unless otherwise stated in Table 2). This was the only habituation to the tests. The following tests were performed in the same order; one leg stand, figure of eight run, handgrip strength, jump and reach, modified push-up, dynamic sit-up, and 2 km walk test. Investigators (3 male; 3 female) were trained to use standardised language and consistent encouragement. A description of the tests performed is provided in Table 2. The best test trial for each measure was used for analyses.

**Table 2:** Detailed description of the ALPHA-FIT field test battery for adults aged 18–69 y performed in the present study. Tests were performed in the order listed.

Test	Procedure	Practice and test trials
One leg stand (maximum test duration is 60 s)	Participants chose their preferred leg to stand on, and placed the heel of the opposite foot at the height of the knee, with their whole foot flat against the medial aspect of the gastrocnemius. Arms were extended at either side of the body, and eyes closed. Timing was started when the participant reached the correct position, and was stopped either when they lost balance (i.e. foot of the free leg lost contact with the supporting leg), or 60 s was reached	Preferred leg was chosen by practising standing on each leg. Two trials were performed unless the result of the first trial was 60 s. The investigator demonstrated correct technique
Figure of eight run	Two cones were set facing each other 10 m apart. The start/finish line was set at the first cone. When the participant heard the command ‘go’ the stopwatch was concurrently started and they ran as fast as possible around the second cone and back around the first cone to the start/finish line. The stopwatch was stopped when they crossed the start/finish line again	One practice trial. Two test trials
Handgrip strength	The electronic hand dynamometer (model EH101, CAMRY, China) was used to measure handgrip strength. Participants stood upright with the dynamometer in their preferred hand. The preferred arm was extended slightly away from the body, with the scale facing the investigator. Participants were instructed to squeeze the handle of the dynamometer as forcefully as possible keeping their arm straight and slightly away from their body, with no extraneous movements	One practice trial. Two test trials (with 10 s rest in-between). The investigator demonstrated correct technique
Jump and reach	The participant stood beside a wall facing forward. Using chalk on their fingers they raised their dominant arm up to measure ‘standing height’. They were then instructed to jump as high as possible using their arms and flexing their knees to enhance their performance. However, their whole foot had to stay on the ground when flexing their knees. Using their middle finger, they were asked to make a chalk mark on the wall while at their highest position. The vertical distance between ‘standing height’ and jump height was measured with a tape measure	One practice trial. Two test trials. The investigator demonstrated correct technique
Modified push-up	Participants lay prone on an exercise mat, and began the push-up cycle by clapping their hands behind their back once. A normal straight leg push-up with elbows completely straight in the up position so that the participants could touch his/her either hand with the other hand was performed. The participant ended the push-up cycle in the prone position and repeated this cycle. Participants were instructed to perform as many push-up cycles as possible in 40 s	The different phases of the modified push-up cycle were practiced once before the test trial. There was only one test trial. The investigator demonstrated correct technique
Dynamic sit-up (maximum number of sit-ups is 15)	Participants lay supine on the exercise mat with knees flexed (90^o^). Participants were instructed to keep their ankles and knees together, and the investigator supported the ankles with his/her hands to keep the feet on the mat throughout the sit-up. Five repetitions of sit-ups were performed in three different hand positions (i.e. levels). The first five sit-ups were performed with the fingertips of both hands reaching the midpatella from a straight lying position while keeping the arms straight and palms resting on the thighs. The second five sit-ups were performed with arms folded over the chest. Participants had to reach their thighs with both elbows. The last five sit-ups were performed with fingertips of each hand around the earlobes. Participants had to reach their thighs with both elbows. No rest was provided between each level. Between each sit-up the back of the head and the elbows had to touch the mat	No practise trials. One test trial. The investigator demonstrated correct technique
2 km walk test	One lap of the sports hall was measured using a measuring wheel, and the corresponding laps calculated. A start and finish line, and cones were used to ensure the participant did not cut any corners. All participants walked anti-clockwise. The investigator counted the laps for the participant. Participants were instructed to walk as fast as possible following the International Association of Athletics Federation racewalking rules (i.e. front knee must not be bent when making contact with the ground, and one part of the foot must remain in contact with the ground at all times). Investigators ensured compliance	One practice trial of 200 m only

#### Statistical analyses

Statistical analyses were performed using the Statistical Package for the Social Sciences (SPSS) version 25 (IBM, SPSS Inc, Chicago, IL, USA) and magnitude-based inferences (MBIs) customizable spreadsheets, using the raw data [55]. Prims8 (GraphPad Software, San Diego, CA, USA) was used to create the figures. Descriptives were checked and confirmed for assumptions of normality using quantile-quantile (Q-Q) plots (Grafen and Hails, 2002). Linear Mixed Models (LMM) were performed to examine the differences in ALPHA-FIT test results and physical self-concept between males and females, and ALPHA-FIT test results and school-based or extra-curricular PA groups. Fixed (gender, school-based or extra-curricular PA) and random (participants) effects for the LMM were fit for each dependent variable (30). For extra-curricular PA, data was analysed separately for males and females. The least squares mean test provided pairwise comparisons between the fixed effects. Step down Hommel p value adjustments were used for post hoc analysis in the event of a significant effect [56]. Normality and homogeneity of variance of the residuals were checked using Q-Q plots, and scatter plots respectively, and deemed plausible in each instance. This type of analysis was preferred as it can accurately model between-subject variability [57–59]. Cohen’s d effect sizes (ES), and 90% confidence limits (CLs) were obtained using the MBI spreadsheets [60], and categorized using standardized thresholds of; < 0.2 trivial, 0.21–0.60 small, 0.61–1.20 moderate, 1.21–2.0 large, and > 2.0 very large [55]. These magnitudes were further interpreted using the following qualitative descriptions; <0.5 most unlikely, 0.5–5% very unlikely, 5–25% unlikely, 25–75% possibly, 75–95% likely, 95–99.5% very likely, and > 99.5% most likely [55]. Differences were considered meaningful if there was a > 75% likelihood of the observed effect exceeding the smallest worthwhile effect (0.20 x between subject SD) for the ALPHA-FIT tests, and half a step of the PSDQ-S Likert scale [61]. The smallest worthwhile change was defined as 0.20 x between subject SD for the ALPHA-FIT tests due to an absence of specific recommendations for this type of data within the existing literature. Data is reported as ES; ±90% CL. Data are reported as mean ± standard deviation (SD). Significance was accepted as p ≤ 0.05.

## Results

BMI and engagement in school-based and/or extra-curricular PA are reported in Table 3.

**Table 3:** Participants BMI and engagement in school-based and extra-curricular physical activity (PA). BMI is reported as a percentage for each category, and as an overall average (mean ± standard deviation).

	Males	Females
BMI < 18 kg∙m^2^	9%	21%
BMI 18–25 kg∙m^2^	56%	55%
BMI 25–30 kg∙m^2^	19%	15%
BMI > 30 kg∙m^2^	16%	8%
Average BMI (kg∙m^2^)	24.5 ± 5.55	22.5 ± 4.62
‘Yes’ to school-based PA	92%	84%
‘No’ to school-based PA	8%	16%
‘Yes’ to extra-curricular PA	77%	35%
‘No’ to extra-curricular PA	23%	65%

BMI = body mass index

Results from the LMM showed there were no differences for school-based PA (p ≥ 0.78) or for extra-curricular PA for males (p ≥ 0.26) or females (p ≥ 0.21) for the ALPHA-FIT test or physical self-concept. Therefore, in line with the principle of parsimony, only comparisons between males and females are reported below.

### Physical fitness

**Fig 1:**
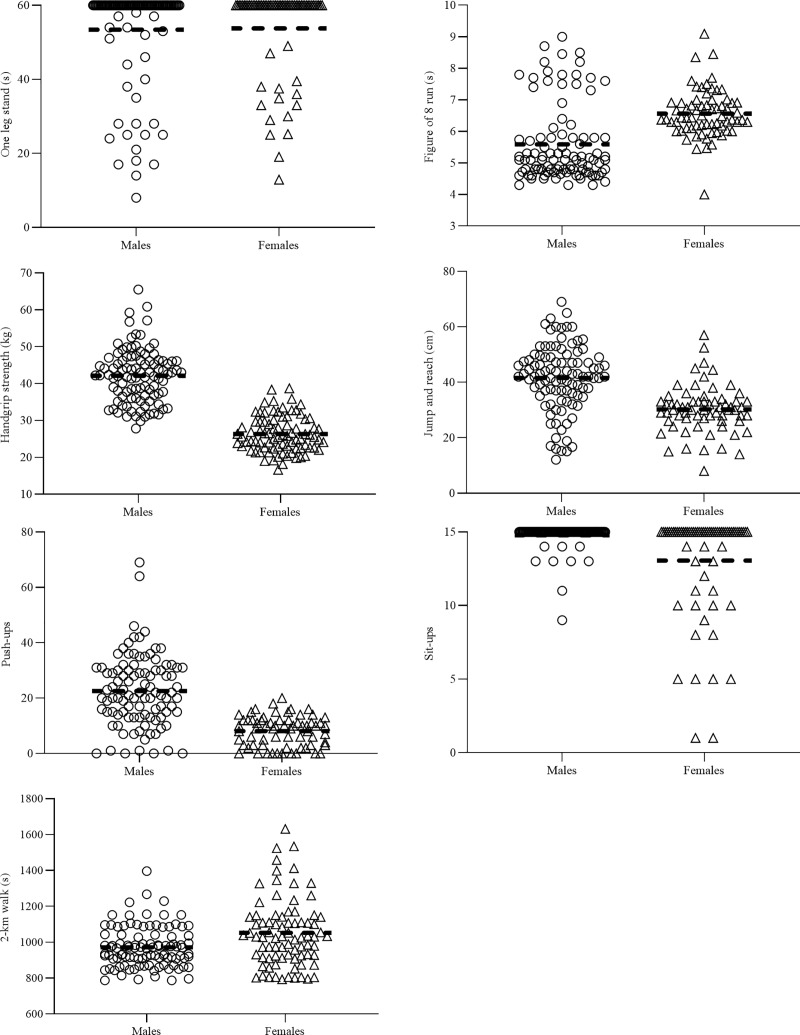
Individual ALPHA-FIT test battery results for males and females. Dashed horizontal line represents the mean.

Substantial differences in ALPHA-FIT test results between males and females were observed (Table 4). Individual results are shown in Fig 1, and average results in Table 4.

**Table 4:** The ALPHA-FIT test results comparison for males and females. Data is presented as mean ± standard deviation. 90% confidence limit and probabilities that the likelihood of the observed effect demonstrated males were fitter, trivial differences, or females were fitter based on 0.2 x between subject SD are presented.

ALPHA-FIT test	Males	Females	LMM (95% CI)	90% CL and probabilities
One leg stand (s)	53.4 ± 13.6	53.7 ± 12.4	p = 0.87, f = 0.01 (4.5 to 3.8)	0.12; ±1.20, trivial **most likely trivial differences** **(0/100/0)**
Fig 8 run (s)	5.6 ± 1.2	6.6 ± 0.7	p = 0.001, f = 37.3 (0.7 to 1.3)	0.86; ±0.42, moderate **very likely males were fitter** **(99/1/0)**
Handgrip (kg)	42.1 ± 11.9	26.3 ± 4.8	p < 0.001, f = 283.6 (14.0 to 17.6)	2.1; ±0.75, very large **likely males were fitter** **(92/8/0)**
Jump and reach (cm)	41.5 ± 11.9	30.2 ± 8.6	p < 0.001, f = 43.8 (8.2 to 14.5)	0.93; ±0.32, moderate **most likely trivial differences** **(0/100/0)**
Modified push-up	23 ± 13	8 ± 5	p < 0.001, f = 74.7 (12 to 17)	0.99; ±0.34, moderate **most likely trivial differences** **(0/100/0)**
Dynamic sit-up	15 ± 1	13 ± 4	p < 0.001, f = 20.5 (1 to 3)	2.2; ±0.76, very large **most likely males were fitter** **(100/0/0)**
2 km walk (s)	972 ± 113	1052 ± 188	p < 0.001, f = 12.7 (33 to 127)	0.65; ±0.16, moderate **most likely trivial differences** **(0/100/0)**

PA = physical activity; CI = confidence interval; CL = confidence limit. Differences are expressed as standardized effect sizes; ±90% confidence limits. Meaningful differences [i.e. > 75% likelihood of the observed effect exceeding the smallest worthwhile effect (0.20 x between subject SD)] are shown in bold.

### Physical self-concept

**Fig 2:**
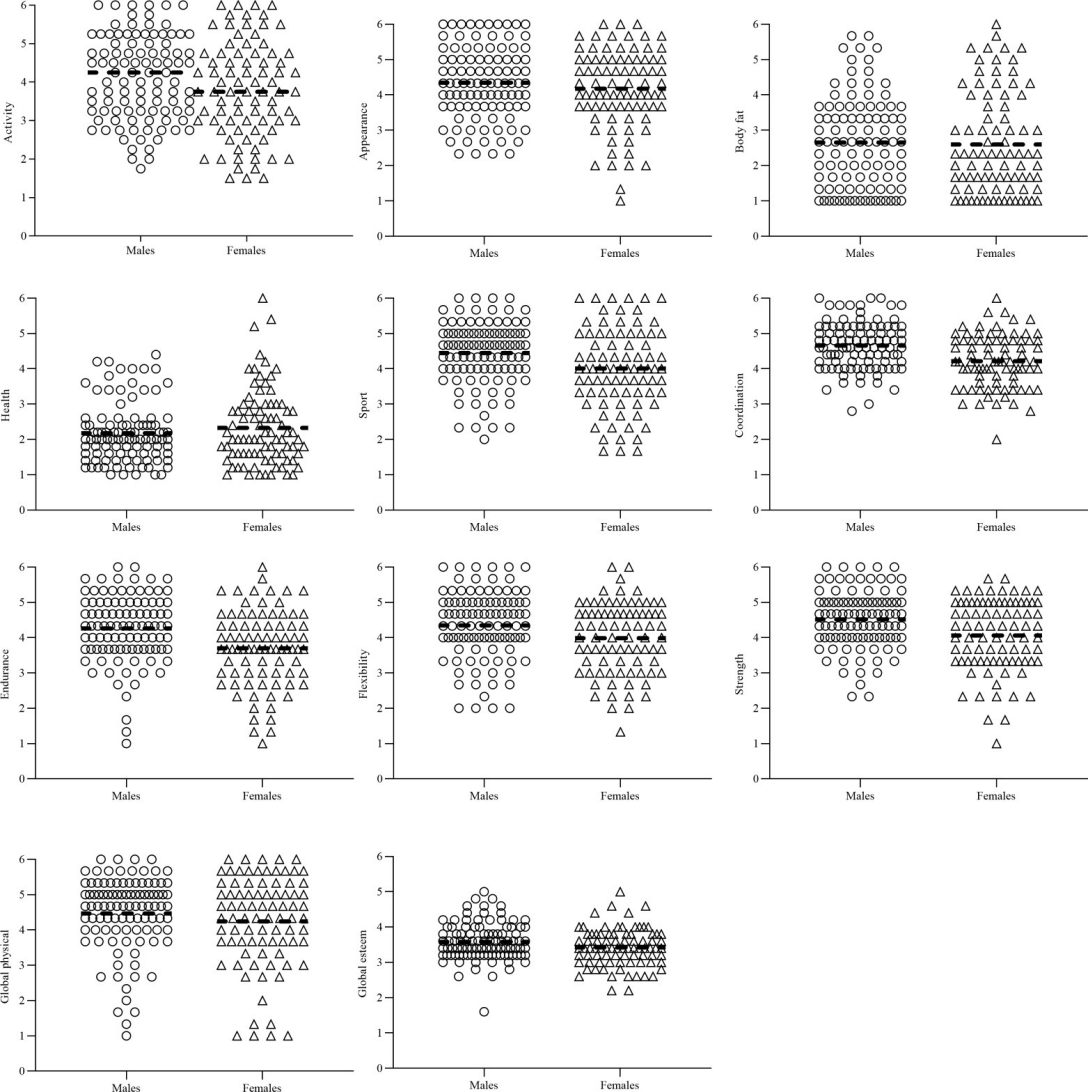
Individual physical self-concept results for males and females measured using the physical self-description questionnaire (PDSQ-S). Dashed horizontal line represents the mean.

Substantial differences in PSDQ-S results between males and females were observed (Table 5). Individual results are shown in Fig 2, and average results in Table 5.

**Table 5:** The physical self-concept physical self-description questionnaire (PSDQ-S) results for males and females. Data is presented as mean ± standard deviation. 90% confidence limit and probabilities that the likelihood of the observed effect demonstrated males scored higher, trivial differences, or females scored higher based on half a step point of the PSDQ-S are presented.

PSDQ component	Males	Females	LMM (95% CI)	90% CL and probabilities
Activity	4.1 ± 1.1	3.8 ± 1.2	p = 0.07, f = 3.4 (-0.03 to 0.66)	0.27; ±0.24, small **likely trivial differences** **(6/94/0)**
Appearance	4.3 ± 1.0	4.2 ± 1.1	p = 0.28, f = 1.2 (-0.14 to 0.48)	0.20; ±0.31, trivial **likely trivial differences** **(5/95/0)**
Body fat	2.7 ± 1.3	2.6 ± 1.4	p = 0.79, f = 0.1 (-0.34 to 0.45)	0.01; ±0.06, trivial **most likely trivial differences** **(0/100/0)**
Health	2.2 ± 0.8	2.3 ± 1.1	p = 0.29, f = -1.1 (-0.43 to 0.13)	0.22; ±0.34, small **likely trivial differences** **(16/84/0)**
Sport	4.4 ± 0.9	4.0 ± 1.1	p = 0.004, f = 8.6 (0.14 to 0.73)	0.49; ±0.28, small possibly males higher (57/43/0)
Coordination	4.7 ± 0.7	4.2 ± 0.8	p < 0.001, f = 17.9 (0.24 to 0.65)	0.78; ±0.37, moderate **very likely males higher** **(99/1/0)**
Endurance	4.3 ± 1.0	3.7 ± 1.1	p < 0.001, f = 14.3 (0.27 to 0.65)	0.66; ±0.27, moderate **likely males higher** **(87/13/0)**
Flexibility	4.3 ± 1.0	4.0 ± 1.0	p = 0.02, f = 6.0 (0.07 to 0.65)	0.30; ±0.21, small **likely trivial differences** **(9/91/0)**
Strength	4.5 ± 0.8	4.1 ± 1.0	p = 0.001, f = 11.4 (0.19 to 0.72)	0.49; ±0.24, small possibly males higher (55/45/0)
Global physical	4.5 ± 1.1	4.2 ± 1.3	p = 0.19, f = 1.7 (-0.11 to 0.58)	0.21; ±0.26, small **likely trivial differences** **(8/92/0)**
Global esteem	3.6 ± 0.5	3.4 ± 0.5	p = 0.04, f = 4.2 (0.01 to 0.31)	0.35; ±0.28, small **likely trivial differences** **(19/81/0)**

PSDQ = physical self-description questionnaire; LMM = linear mixed model; CI = confidence interval; CL = confidence limit. Differences are expressed as standardized effect sizes; ±90% confidence limits. Meaningful differences [i.e. > 75% likelihood of the observed effect exceeding the smallest worthwhile effect (half step point)] are shown in bold.

## Discussion

This is the first study within Qatar to obtain gender stratified objective measures of physical fitness. The main findings showed on average males were observed to have greater power, strength and speed compared to females (Table 4). Endurance and coordination physical self-concept, on average was higher for males (Table 5). Anthropometry for our participants (Tables 1 and 2) was similar to previously published data of university students in Qatar [62], which reported that 34% males and 23% females were classified as overweight or obese.

Physical fitness results in the present study (Table 4) are lower than worldwide (e.g. Australia, UK, USA, Canada, Norway) age and gender stratified normative data for handgrip strength [37, 38] and push-up capacity [44, 47]—see Table 6 for direct comparisons. However, caution should be taken when comparing these results as different handgrip dynamometers [37], handgrip procedures (e.g. hand used, flexed elbow) [38], and push-up capacity test differences (e.g. modified v strict push-up) [45] compared to our study [53] will affect interpretation of the findings. Therefore, future studies should employ standardised procedures when possible. For example, the standard handgrip procedure for clinical assessment recommendations proposed by the American society of hand therapists should be followed to allow direct comparison with normative data [63]. Very limited physical fitness data is available for the Middle East [64], with no such data available for Qatar. Therefore, whilst the present data is a welcome addition to the global picture, the current absence of data from the immediate region, precludes geographically local comparisons. To contextualise, average handgrip strength for males (42.3 kg) and females (25.6 kg) in the present study was above the clinical cut-off for ‘muscle weakness’ in UK adults [30]. Furthermore, despite modified push-ups (i.e. easier than standard push-ups) being performed in our study [53] on average the total number of push-ups performed by males (n = 22), is significantly lower than the suggested number required (n = 40) to attenuate cardiovascular disease event risk [29]. Subsequently, these simple low-cost measures (i.e. handgrip strength and push-ups) could have clinical utility within Qatar.

**Table 6:** Handgrip strength and push-up capacity normative values in young adults worldwide compared to young Qatari adults (17–26 y) in the present study. Data is presented as mean ± standard deviation.

Age stratified results	Sample size	Males	Females	Qatar male difference (n = 101)	Qatar female difference (n = 85)
Handgrip strength (kg)					
Australia (20–29 y) (23)	m = 17, f = 12	47 ± 10 (R), 45 ± 9 (L)	30 ± 7 (R), 28 ± 6 (L)	- 4.7 (R), -2.7 (L)	-4.4 (R), -2.4 (L)
USA (20–24 y) (25)	m = 74, f = 80	54 ± 10 (D), 50± 10 (ND)	31 ± 6 (D), 29 ± 6 (ND)	-11.7 (R), -7.7 (L)	-5.4 (R), -3.4 (L)
UK (20–24 y) (28)	m = 10, f = 10	48 ± 8 (R), 43 ± 8 (L)	28 ± 4 (R), 26 ± 6 (L)	-5.7 (R), -0.7 (L)	-2.4 (R), -0.4 (L)
Norway (20–29.9 y) (29)	m = 40, f = 36	58 ± 10	33 ± 6	-15.7	-7.4
Push-up capacity					
USA (20–29 y)(30)	f = 15	n/a	17 ± 7	n/a	-7
USA (18–22 y) (32)	m = 37, f = 46	19 ± 4	12 ± 3	+3	-2
Norway (20–29.9 y) (29)	m = 39, f = 23	14 ± 3	10 ± 4	+8	0

Y = years; m = males, f = females, R = right; L = left, D = dominant, ND = non-dominant

The gender stratified physical fitness results (Table 4) are similar to worldwide data (e.g. Switzerland, Chile), which typically shows poorer cardiorespiratory and muscular fitness for females compared to males [65–67]. Indeed, in Qatar physical inactivity is more prevalent in females throughout childhood and adulthood [4, 48, 62, 68], likely due to cultural barriers. Given the relationship between PA and physical fitness, poorer performance in the ALPHA-FIT test results in our study (i.e. figure of eight, handgrip, dynamic sit up) for females, may be partially explained by higher physical inactivity for Qatari females.

Engagement in school-based PA was similar between males and females (Table 1), however, extra-curricular PA was much higher for males (77%), compared to females (35%). School-aged PA appears to be associated with adult PA [23], therefore, insufficient PA during childhood, could explain the poor results obtained in the present study. Indeed, within Qatar only 39% of children (6–12 y) met the school-based moderate-to-vigorous PA of ≥ 30 min per day [4], reflective of the ‘D’ grade obtained for PA in the Qatar Active Healthy Kids Report Card [5], significantly below other countries [6]. This may explain why there was no difference in physical fitness for those who stated they engaged in school-based PA in our study. Despite this there is a lack of objective data on the physical fitness of Qatari children [5]. Subsequently, an age and gender stratified physical fitness database representative of the Qatari population should be established.

The physical self-concept results we observed show that on average the participants had positive physical self-concept for all components (Fig 2). However, the variability of the data demonstrates that several participants scored negatively for some components, particularly body fat (Fig 2). On average males *likely* had ‘moderately’ higher physical self-concept for coordination and endurance, (Table 5). Similarly, male Iranian university students scored significantly higher for coordination, but also for health, body fat, global physical and global esteem physical self-concept components compared to females [50]. As shown in our data higher physical self-concept is associated with greater engagement in PA [51], and enhanced motor skill development [52]. Whilst the present study design cannot establish cause-and-effect, lower physical self-concept may help explain why females performed worse than males in the ALPH-FIT test. However, future research would be required in order to ascertain this. Low physical fitness and physical self-concept for females in the present study is likely a complex interaction of socio-ecological factors [69]. For example, Islamic traditional clothing (i.e. Abaya, Hijab), adopted widely by Qatari females in public places, has been considered an additional barrier regarding PA engagement [49]. Female students previously reported they did not like to wear sports clothes underneath their Abayas [70]. Additionally, women traditionally need to be accompanied by a male family member when going outdoors [69]. Furthermore, the normalization of increased post-puberty weight [71, 72], and a male preference for a ‘heavier’ shape in Arab women [73], could decrease physical self-concept. Evidently, Qatar is attempting to raise the profile of Qatari females in sport to inspire females to be more active [74], nevertheless females still face additional barriers and challenges to PA engagement, compared to males. Subsequently, gender-specific PA interventions for children and adults, which increase physical fitness are necessary to decrease early morbidity and mortality in this population.

Caution must be taken when interpreting these results, as the convenience sample was homogeneous, and therefore, these results may not be representative of the Qatari population as a whole. Additionally, all investigators were from the Sport Science Program at Qatar University, and therefore, the authors speculate that those who volunteered for the study were the more ‘health conscious’ students from Qatar University. Subsequently, it would be beneficial to administer physical fitness tests across all schools and workplaces within Qatar, to establish a comprehensive and accurate database of the Qatari population. Nevertheless, the utilisation of a field best test (i.e. ALPHA-FIT test battery), is a strength of this study as the measures are time-efficient, of low-cost, and can be easily administered with little equipment. However, there is currently no data examining the validity and reliability of the ALPHA-FIT test battery for adults, and therefore future research should either measure the test-retest reliability and/or use validated and reliable alternatives. It was clear from our results that the one leg stand (maximum 60 s), and dynamic sit-up test (maximum 15 reps), may not have been appropriate, given the majority of participants were able to reach the maximum performance for these tests (Fig 1). Alternative balance and dynamic strength tests should be employed in future investigations.

## Conclusion

This is the first study to obtain objective measures of physical fitness within Qatar. Overall, the results appear lower than worldwide stratified normative data, and in agreement with previous research showing higher physical fitness and physical self-concept in males compared to females. The reasons for higher physical inactivity, and lower physical self-concept and physical fitness in females in particular, is presently unclear. Future research should focus on elucidating the aetiology of this, in order to enhance PA engagement, subsequently improving physical fitness, and reducing morbidity and mortality.

## Supporting information

S1 Data Raw data ALPHA FIT and PSQD-S. (XLSX)
